# Capsaicin, Nociception and Pain

**DOI:** 10.3390/molecules21060797

**Published:** 2016-06-18

**Authors:** Bárbara Frias, Adalberto Merighi

**Affiliations:** 1 Department of Integrative Medical Biology, University of Umea, 901 87 Umea, Sweden; Barbara.F.Frias@umu.se; 2 Department of Veterinary Sciences, University of Turin, Largo Paolo Braccini 2, I-10095 Grugliasco (TO), Italy

**Keywords:** capsaicin, vanilloids, TRPV1 receptor, nociception, somatic pain, visceral pain, sensitization, analgesia, resinferatoxin

## Abstract

Capsaicin, the pungent ingredient of the hot chili pepper, is known to act on the transient receptor potential cation channel vanilloid subfamily member 1 (TRPV1). TRPV1 is involved in somatic and visceral peripheral inflammation, in the modulation of nociceptive inputs to spinal cord and brain stem centers, as well as the integration of diverse painful stimuli. In this review, we first describe the chemical and pharmacological properties of capsaicin and its derivatives in relation to their analgesic properties. We then consider the biochemical and functional characteristics of TRPV1, focusing on its distribution and biological effects within the somatosensory and viscerosensory nociceptive systems. Finally, we discuss the use of capsaicin as an agonist of TRPV1 to model acute inflammation in slices and other *ex vivo* preparations.

## 1. Introduction and General Concepts

### 1.1. Chemical Features of Capsaicin

Capsaicin (trans-8-methyl-N-vanillyl-6-nonenamide—C_18_H_27_NO_3_) is a naturally occurring substance derived from the plants of the genus *Capsicum*, family *Solanaceae*. Capsaicin is a vanilloid as it contains a vanillyl group in its formula. Vanilloids belong to a class of organic chemicals referred to as protoalkaloids, *i.e.*, alkaloids where nitrogen is located in the side chain. Alkaloids are a wide and rather heterogeneous group of compounds that are generally made of carbon, hydrogen and nitrogen. In addition to these, alkaloids often contain other elements among which is oxygen, as is the case for capsaicin. The first isolation of the vanilloid from paprika and cayenne dates back to 1876 and was reported by Thresh [1]. The first study on capsaicin structure dates back to 1920 [2].

The commercial production of capsaicin from natural sources primarily involves its isolation from *Capsicum* spp. Capsaicin can also be produced by the reaction of vanillylamine with 7-methyloct-5-ene-1-carboxylic acid chloride. The vanilloid appears in the form of a highly volatile, pungent, hydrophobic, colorless and odorless white crystalline powder. Once absorbed by the body, capsaicin is likely metabolized by dehydrogenation, giving rise to specific macrocyclic, -diene and -imide metabolites.

### 1.2. Natural Sources of Capsaicin

In the world, there are five known domesticated varieties of *Capsicum* spp.: *C. annuum*, *C. frutescens*, *C. chinense*, *C. baccatum* and *C. pubescens*. *C. annuum* (also known as red chili, paprika, gendot, curly chili) and *C. frutescens* (rawit) are very similar, to the point that some authors do not consider the two as different species [3]. The fruit of all these plants has a hot taste that derives from its content in capsaicinoid compounds: a group of amide acids from vanilinamine and fatty acid chain branched at C9 and C11. Capsaicinoids in *Capsicum* spp. for the most contain capsaicin, and, in lesser quantities, dihydrocapsaicin, homocapsaicin and homodihydrocapsaicin. Analysis of the levels of capsaicin in various *Capsicum* fruits showed that green paprika, yellow paprika and red paprika contained no capsaicin, while chili tanjung, red chili, red gendot, green gendot, green curly, japlak rawit, red curly, red rawit and green rawit (cayenne) contained 0.38; 0.83; 0.87; 0.88; 1.05; 1.09; 1.14; 1.85 and 2.11% capsaicin (w/w), respectively [4].

### 1.3. Cloning, General Distribution, Functional Properties and Biological Effects of the Capsaicin Receptor in Mammals

#### 1.3.1. Cloning and General Distribution of TRPV1

The capsaicin receptor, named transient receptor potential vanilloid 1 receptor (TRPV1), was cloned in 1997 from rat dorsal root ganglia (DRGs) using a functional screening strategy for isolating candidate complementary DNA (cDNA) clones [5]. This newly cloned cDNA was initially named VR1, for vanilloid receptor subtype 1. Later, VR1 was identified to be a member of the transient receptor potential (TRP) family of cation channels and the nomenclature TRPV1 was adopted to denote this association. To date, TRPV1 has been cloned from human, guinea pig, rabbit, mouse and porcine tissues. Its distribution was mainly investigated in tissues and organs from human, rat and mouse, but also several other mammals among which are the other aforementioned species [6]. By reverse transcription-polymerase chain reaction (RT-PCR), TRPV1 was localized to human DRGs, brain, kidney, pancreas, testis, uterus, spleen, stomach, small intestine, lung and liver [7]. In rats, with an array of techniques including *in situ* hybridization, northern blotting, RT-PCR and immunocytochemistry (ICC), the receptor was localized in numerous areas of the central nervous system (CNS) including the cerebral cortex, striatum, hippocampus, central amygdala, thalamus, hypothalamus, cerebellum, locus cerulean, cochlear nuclei, spinal nucleus of the trigeminal nerve (SNTN), inferior olive and spinal cord [8]. In the peripheral nervous system (PNS), TRPV1 was detected in rat trigeminal ganglion (TG) and DRGs. Other rat organs expressing the receptor were the kidney, pancreas, placenta and urinary bladder [5,7,9]. In mice, TRPV1 was localized to similar districts of the CNS and PNS than in rats [10,11,12,13,14]; and a subset of smooth muscle cells in small arteries [15]. In the above localizations and species, TRPV1 was not only detected in intramural nerve fibers and plexuses, providing the visceral innervation to the organs and tissues listed above, but also in the mucosal epithelial cells. The latter are not the only non-neural cells expressing the receptor, as some cells of the immune system, e.g., the T-cells and the mast cells; the keratinocytes of the epidermis; the cells of inner root sheet and the infundibulum of hair follicles; differentiated sebocytes; the cells of sweat gland ducts and the secretory portion of eccrine sweat glands; and the vascular endothelium also express TRPV1 [16].

#### 1.3.2. Functional Properties and Biological Effects of TRPV1

Initial studies on isolated cells demonstrated that capsaicin and other natural substances, as well as some physical activators and protons, activated TRPV1. Functionally, capsaicin, resinferatoxin (RTX) and heat activated Human Embryonic Kidney 293 (HEK 293) cells transfected with human or rat TRPV1 vector [5,7]. Mouse DRG neurons were activated by the same substances in patch-clamp whole- or single-cell recordings [17]. Capsaicin and acidic pH in *Xenopus laevis* oocytes injected with the human TRPV1 cDNA [7] effectively opened the receptor channel in two-electrode voltage clamp experiments. In addition, intracellular Ca^2+^ imaging provided further evidence that the receptor was activated by capsaicin, anandamide, olvanil, RTX and pH in HEK 293 cells transfected with rat [18], mouse [19] or human [20] TRPV1 cDNA. In neurons, cation (Ca^2+^) influx through TRPV1 causes membrane depolarization, leading to the activation of voltage-gated sodium channels and the generation of an action potential. It was very recently reported that the capsaicin-evoked action potentially follows a physical interaction between TRPV1 and anoctamin 1, a calcium-activated chloride channel, resulting from the entry of Ca^2+^ through the TRPV1 pore and that such interaction is relevant for the enhancement of nociception [21].

Broadly speaking and in the more complex *in vivo* or *ex vivo* context, TRPV1 has been linked to thermo-sensation (heat), autonomic thermoregulation, nociception, food intake regulation and multiple functions in the gastrointestinal (GI) tract [22]. Specifically in CNS, the receptor has also been involved in growth cone guidance, long-term depression, endocannabinoid signaling and osmosensing, the latter by a particular TRPV1 variant [16].

Notably, TRPV1 is also up regulated in several human pathological conditions including vulvodynia [23], GI inflammation, Crohn’s disease and ulcerative colitis [24,25].

In the light of the aforementioned observations, it is not surprising that capsaicin has been clinically used and proven to be of some benefit in obesity, cardiovascular and GI pathologies, various types of tumors, neurogenic bladder and certain dermatologic conditions, although many of these pharmacological effects appeared to be TRPV1-independent [22].

#### 1.3.3. Other Molecular Targets of Capsaicin

Some of the TRPV1-independent pharmacological effects have led a debate on the possible existence of other receptor targets of capsaicin. There is contrasting evidence regarding the possibility that capsaicin may be an inhibitor of signal transducer and activator of transcription 3 (STAT3), although the lowest active dose (50 μM) necessary to inhibit STAT3 is significantly higher than the concentration (1–5 μM) usually required to stimulate TRPV1 [26].

It seems also possible that—at least under certain conditions—neurokinin 1 (NK1) and neurokinin 2 receptor-mediated mechanisms are involved in the induction of phosphorylated extracellular signal-regulated kinase (pERK) after stimulation of primary sensory neurons (PSNs) with capsaicin, an effect not solely directly linked to the binding of the vanilloid to TRPV1 channels [27]. However, as PSNs release substance P in response to capsaicin challenge, such a possibility still needs to be confirmed in full.

Very recently, additional potential targets of capsaicin were predicted by reverse docking and confirmed via chemical-protein interactome and molecular docking. By this approach capsaicin was identified as an inhibitor of carbonic anhydrase 2 [28].

### 1.4. Nociception and Pain

Nociception is the encoding of a noxious stimulus *i.e.*, an actual or potential tissue damaging event [4] and its transduction into electric signals. Noxious stimuli are detected by nerve endings found throughout the body and originating from the PSNs, which represent the first element of a polyneuronal chain leading to the perception of pain. A class of PSNs, the nociceptors, respond to nociceptive stimuli but under certain conditions can be activated also by innocuous forms of the same stimulus e.g., in the case of heat and cold nociceptors. Nociceptors are polymodal receptors as they respond to stimuli of a heterogeneous nature: mechanical (e.g., high pressure), thermal (too high or too low temperatures) and chemical.

Once activated by an adequate stimulus, nociceptors in skin, muscles, joints or viscera generate a nerve signal (action potential) that is ultimately transferred to the somatosensory cortex, the parieto-insular cortex and to the anterior cingulate cortex where the sensation of pain is perceived. Nociceptive stimuli conveyed from somatic and visceral organs to cortical centers follow different routes. In particular, the trigeminal and spinal nerves solely provide somatic sensory fibers to the skin and the organs of locomotion, whereas most viscera have two distinct types of sensory nerves originating from the sympathetic (via the white communicating branches of the thoracic and lumbar nerves) and parasympathetic (via certain cranial nerves) divisions of the autonomic nervous system.

### 1.5. Basic Organization of Somatic and Visceral Pain Pathways

#### 1.5.1. Somatic Pain Pathways

**Figure 1:**
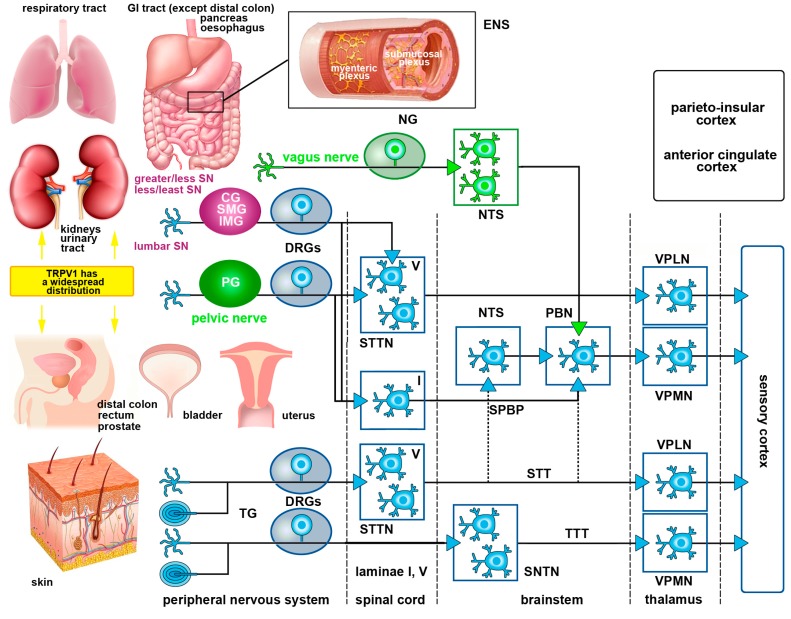
Illustration depicting TRPV1 receptor distribution in body organs and the main visceral and somatic pain pathways. TRPV1 is found in several body organs and when its expression increases, it contributes to the development of visceral and somatic pain. Afferent fibers innervating the viscera project to the CNS following the course of autonomic sympathetic and parasympathetic nerves. Afferent sympathetic fibers originating from the thoracolumbar DRGs follow the hypogastric, lumbar and splanchnic nerves (SN) after traversing the sympathetic prevertebral (celiac ganglion—CG, superior mesenteric ganglion- SMG and inferior mesenteric ganglia—IMG) and paravertebral ganglia (in magenta). Ascending projections from lamina I neurons in the spinal cord travel along the spinoparabrachial pathway (SPBP) to the parabrachial nucleus (PBN), whereas projections from deep dorsal horn neurons (STTN) travel along the spinothalamic tract (STT) to thalamic nuclei (VPMN and VPLN). The parasympathetic sensory innervation (in green) follows the vagus and pelvic nerve that terminate in the brainstem and lumbosacral cord, respectively. The gastrointestinal tract also has its own autonomic intrinsic nervous system—the enteric nervous system (ENS) — constituted by the submucosal (Meissner’s) and myenteric (Auerbach’s) plexuses. Enteric plexuses play a key role for communication with the autonomic extrinsic nervous system in several GI functions. Nociceptive somatic inputs from all the parts of the body, except the head, are transmitted to spinothalamic projection neurons in the dorsal horn of the spinal cord. These neurons in turn, reach the neurons in the ventro-postero lateral nucleus of the thalamus (VPLN) through the STT. Nociceptive somatic inputs (for simplicity, only the skin is depicted but these inputs also derive from the muscles, tendons, bones and joints) from the head are relayed to the spinal nucleus of the trigeminal nerve (SNTN) and then, along the trigeminothalamic fibers to the ventro-postero medial nucleus of the thalamus (VPMN). Finally, nociceptive input is transferred to the sensory cortex where it is perceived as pain. Affective, emotional and autonomic aspects of pain are processed in other cortical areas (black).

Somatic pain pathways collect stimuli from the skin, muscles, joints, ligaments and bones. With the exception of the head and the proximal regions of the neck which are innervated by the peripheral projections of the trigeminal PSNs, nociceptive stimuli from all other parts of the body are encoded by the PSNs of the DRGs. The main somatic pain pathways are the trigeminothalamic and the spinothalamic pathways. They are critical for the sensory discriminative aspects of pain perception. Both consist of a polysynaptic chain of three main neurons, often referred to as first, second and third order somatic sensory neurons. First order sensory neurons (the PSNs) are located in the TG, the proximal ganglia of the glossopharyngeal and vagus nerves, or the DRGs. Second order sensory neurons lie in the spinal cord dorsal horn, or the SNTN. These projection neurons give rise to axons that cross the midline and travel ascending all along the spinal cord and brainstem to reach the thalamus. Finally, third order sensory neurons reside in the ventral postero-lateral nucleus of the thalamus and eventually send their axons to the cerebral cortex (Figure 1). Modulation of nociceptive signals can occur all along this polyneuronal chain, primarily at the level of the substantia gelatinosa of the spinal cord dorsal horn or the SNTN and by several descending pathways that exert inhibitory or facilitatory effects onto the trigeminothalamic or the spinothalamic neurons.

Other ascending pathways are also important for the general dimension of pain and pain control, as they convey stimuli related to motivational and cognitive aspects (spinoreticular tract and spinoparabrachial tract), motor responses and affectivity (spinomesencephalic tract and spinoparabrachial tract) and neuroendocrine/autonomic responses (spinohypothalamic tract). Notably, the spinoparabrachial pathway is an important route of convergence of somatic and visceral nociceptive stimuli (see Section 1.5.2). For a general description of all these pathways, see [29].

#### 1.5.2. Visceral Pain Pathways

Visceral pain arises from the internal organs including the heart and vessels, airway structures, GI tract and urinary and reproductive organs [30,31]. However, visceral pain is not evoked from all viscera and it is not always linked to a factual visceral injury [32,33]. The diffuse nature and difficulty in locating visceral pain is due to a relatively low density of visceral sensory innervation and extensive divergence of visceral input within the CNS. While in somatic pain, nociceptive C-fibers make synaptic contacts with second-order projection neurons in the upper laminae of the dorsal horn spinal cord and the substantia gelatinosa (lamina II) interneurons [34], input from the viscera has a more diffuse and less topographic distribution and specifically, reaches lamina I and the deep dorsal horn [31,35]. In patients, pain from different visceral organs can have differing areas of presentation, e.g., bladder to perineal area or heart to left arm and neck. Therefore, visceral pain is often localized to distant structures and thus, known as referred pain. Development of symptoms may entail referred pain to somatic structures within the same metameric field as the affected viscera [33]. In addition, secondary hyperalgesia of superficial or deep body wall tissues might occur due to viscerosomatic convergence [32,33,36,37]. Finally, visceral pain is often associated with marked motor and autonomic reflexes such as profuse sweating, nausea, vomiting, GI disturbances and changes in body temperature, blood pressure and heart rate [32,33,36,38,39].

Primary afferent fibers innervating the viscera project to the CNS through autonomic sympathetic and parasympathetic nerves. The sympathetic innervation follows the hypogastric, lumbar and splanchnic nerves which traverse both prevertebral and paravertebral ganglia, until they reach the thoracolumbar region of the spinal cord. The parasympathetic innervation is mediated by vagal and pelvic afferents that terminate in the brainstem and lumbosacral cord, respectively [40,41].

Visceral pain is primarily signaled by spinal afferents, while vagal afferents signal non-painful sensations such as hunger, satiety, fullness and nausea [39]. Although, vagal afferents may not signal pain directly, several studies have documented that stimulation of the vagus nerve attenuates somatic and visceral pain [39]. Vagal afferents originate from PSNs in nodose ganglion that project to the nucleus tractus solitarius (NTS) in the brainstem. Spinally-converging visceral afferents instead, synapse onto the second order neurons within the dorsal horn, which in turn project to higher centers through the dorsal column pathway, the parabrachial pathway and the spinothalamic tract (STT) [31]. Superficial dorsal horn projections, mostly form the spinoparabrachial pathway [42], are associated with autonomic and affective responses to painful stimuli [43]. Along with NTS projections from vagal afferents, spinoparabrachial projections are transmitted to limbic and cognitive higher relay centers including those parts of the brain involved in affectivity, such as the amygdala, hypothalamus and periaqueductal grey (PAG) [31,43]. Visceral spinothalamic projections travel contralaterally from the deep dorsal horn to the main thalamic projections located in the ventroposterior medial and lateral nuclei of the thalamus. The medial thalamic nuclei eventually project to the areas of the prefrontal cortex that are correlated with visceral pain [44,45,46] (Figure 1).

Furthermore, the GI tract besides its extrinsic innervation as mentioned above, has its own intrinsic nervous system, the enteric nervous system (ENS), composed by the submucosal (Meissner’s) and myenteric (Auerbach’s) plexuses [47]. Myenteric plexus lies between the longitudinal and circular muscle layers, whereas the submucosal plexus is found in the submucosa and relays sensory and motor responses to the myenteric plexus, prevertebral ganglia and spinal cord [47,48]. The ENS works autonomously but the digestive function requires communication with the parasympathetic and sympathetic innervations (Figure 1). The ENS contains sensory peptides, primary afferent neurons, interneurons and motor neurons and together with the extrinsic system, controls motor activity, secretion, absorption, blood flow and interactions with other organs such as the pancreas or gallbladder. This way, the gut provides sensory information to the CNS and the CNS can affect GI function [47,48].

### 1.6. Capsaicin as an Analgesic Medication

There is widespread use of capsaicin and other capsaicinoids in traditional medicine, not only in pain therapy but also in body temperature regulation, anti-obesity treatments, anticancer therapy and as antioxidant and antimicrobial agents [49]. In several South-American, African and Asian countries, the leaves and fruits of *Capsicum* spp. have long been in use in the treatment of painful menses, toothache and muscle pain [50]. After the discovery of TRPV1, capsaicin and numerous other natural and synthetic receptor agonists have attracted the attention of the academic and pharma community for their potential in the relief of chronic pain [22]. In national pharmacopoeias, topical capsaicin medications are registered for the treatment of painful states derived from neuralgia, diabetic neuropathy, osteoarthritis and rheumatoid arthritis. Capsaicin-based preparations are also in use to treat pain due to pruritus, psoriasis, mastectomy and bladder disorders.

## 2. The Capsaicin Receptor in Nociceptive Pathways

### 2.1. Structure and Physiology of the Capsaicin Receptor 

#### 2.1.1. Structure and Splice Variants of TRPV1

The painful sensations induced by capsaicin are consequent to its binding TRPV1. As mentioned in Section 1.3.1, TRPV1 is part of the TRP multigene superfamily that encodes a wide number of integral membrane ion channel proteins [16]. Ligand binding and activation profiles of these receptors are closely related but unique to each TRPV subfamily member. The formation of multimeric species [51] and/or heteromultimers among members of the family may increase their functional diversity. For example, TRPV1 and TRPV2 are co-expressed in the IV-VI layer neurons of the adult rat cerebral cortex, with occurrence of multimeric receptor complexes upon receptor activation *in vitro* [52].

Alternative splicing also occurs in the TRP gene family, expanding the number of functionally distinct TRP proteins and potentially providing tissue-specific regulation. Several TRPV1 splice variants have been reported in mice, rats and humans [53,54,55]. While the biological roles of these variants are unclear, the reported deletions lead to nonfunctional channels (mTRPV1b, VR.5'sv and TRPV1VAR) or channels with distinct properties, when examined in recombinant or native expression systems [56]. TRPV1α and TRPV1β are two cDNA variants of TRPV1 as a result of alternate splicing. TRPV1β is a dominant-negative regulator of TRPV1 responses, since it is not functional by itself but inhibits TRPV1α function during co-expression. The recently identified splice variant of the TRPV1 molecule, TRPV1b, produces a negative-dominant effect on the responsiveness of the TRPV1 channel which is increased by peripheral inflammatory processes. TRPV1b has been cloned from mouse DRG neurons, human and rat cDNA libraries respectively. TRPV1b is not sensitive to capsaicin and protons but responsive to heat. Furthermore, TRPV1b but not TRPV1, is expressed on sensory neurons which respond to heat but are not activated by capsaicin. Moreover, when co-expressed with TRPV1, TRPV1b reduces the responses to TRPV1 channel activators in a ratio-dependent manner [57].

#### 2.1.2. Biochemistry and Physiology of TRPV1

TRPV1 is a non-selective cation channel that, similarly to all TRPVs, prefers calcium. The receptor is activated by capsaicin and noxious temperatures with a threshold *in vitro* of approximately 43 °C [5].

The threshold of activation *in vitro* suggests that TRPV1 is inactive at normal body temperature. However such a threshold is dynamically regulated and significantly lowered during inflammation [22]. TRPV1 is also activated, among others, by moderate heat, protons and anandamide, an endogenous ligand of both vanilloid and cannabinoid receptors [5,58]. The full list of natural substances to date reported to be capable of activating/sensitizing the receptor, aside from capsaicin, is reported in Table 1. To these, numerous other synthetic compounds should be added [59].

Like other TRP channels, TRPV1 is a putative six-transmembrane-spanning protein with a pore region localized between transmembrane segments 5 and 6 [5,60]. Numerous studies have led to the identification of the receptor regions and key amino acids involved in specific functions (multimerization, capsaicin action, proton action, heat activation, desensitization, permeability and phosphorylation and modulation by lipids and ATP) [61].

Activity of TRPV1 is positively regulated inside the cell after phosphorylation [5], as its N-terminus has several phosphorylation sites for protein kinases, among which is the calcium and calmodulin-dependent protein kinase II (CaMK II kinase). Some activators listed in Table 1, such as prostaglandins, bradykinin and prokineticin, modulate the activity of the receptor indirectly by activating several different protein kinases inside the cell [61]. Cleavage of phosphatidylinositol 4,5-bisphosphate (PIP2) by phospholipase C (PLC) is another intracellular activation pathway [62,63,64]. Besides PIP2, other negative regulators are intracellular calcium and calmodulin [61]. Instead, phosphatases desensitize TRPV1 after its dephosphorylation [65].

Depending on functional conditions, once activated TRPV1 may become sensitized or enter a refractory state. Studies on these phenomena were mainly carried out *in vitro* and their relevance *in vivo* still needs confirmation for the development of new medications. On cultured human respiratory epithelial cells, sensitization of TRPV1 receptors by antagonists’ pretreatment was shown to be a consequence of the translocation of existing receptors from the endoplasmic reticulum to the cell surface [66]. As an additional possibility, once activated by capsaicin, TRPV1 enters a long refractory state [67]. Refractoriness is a consequence of conformational changes in the receptor protein that depend on extracellular calcium and ultimately, close the channel pore [68].

It should be also mentioned that there seems to exist a marked difference in pharmacological responses between TRPV1 and native capsaicin receptors for the presence of regulatory proteins associated with TRPV1 *in vivo*, among which is Fas-associated factor 1 (FAF1). Notably, FAF1 was demonstrated to constitutively modulate the sensitivity of TRPV1 to various noxious stimuli in sensory neurons by forming an integral component of the vanilloid receptor complex [69].

### 2.2. Localization and Activation of TRPV1 in Pain Pathways

#### 2.2.1. PSNs and Non-Neural Cells

##### Activation of TRPV1 during Neurogenic Inflammation of Skin and Mucosae

The sensitivity of TRPV1 to numerous physical and chemical activators and its widespread distribution indicate that the receptor may be crucial to the onset of inflammation. Activation of TRPV1 on the terminal endings of the sensory fibers derived from somatic and visceral PSNs and some non-neural cells (see Section 1.3.1) produces a calcium and sodium influx that ultimately results in the release of a cocktail of neuropeptides. Among these, the tachykinins substance P and neurokinin A (NKA) and the calcitonin-gene-related-peptide (CGRP) are of particular relevance. Epithelial, endothelial and smooth muscle cells, as well as resident immune cells, respond to these neuropeptides and give rise to a complex series of events collectively referred to as neurogenic inflammation. The main features of neurogenic inflammation, *i.e.*, redness, swelling and pain, are a consequence of the vasodilation, plasma extravasation and hyperalgesia that follow binding of NKA, substance P and CGRP to their cognate receptors on the endothelial and smooth muscle cells [70]. In response to the release of these neuropeptides, mast cells, epithelial cells and immune cells release pro-inflammatory cytokines (e.g., IL1b, IL6, IL8, and TNFα) further contributing to the maintenance of the inflammatory state. The central role played by TRPV1 in the initiation and modulation of neurogenic inflammation suggests that the same factors which influence its functional expression or numbers, could also influence the organism’s response to inflammatory xenobiotics [71].

##### Other Effects of TRPV1 Activation in PSNs

At first, intradermal capsaicin injection into the plantar skin of the hind paw in mice contributes to the development of thermal and mechanical hyperalgesia [5]. Capsaicin also evokes a mechanical allodynia that could be reversed by both G protein and protein kinase inhibitors [90]. At the cell level, the activation of protein kinase A and C evoked by capsaicin results in the phosphorylation of the NMDA receptor subunit NR1 at Ser890/897 and Ser896 respectively, which could be blocked with specific inhibitors [91]. A role for protein kinase B (PKB)/Akt in DRG neurons was also demonstrated using the intradermal capsaicin model [92].

As a consequence of these cellular changes, not only the activation of TRPV1 releases NKA, substance P and CGRP from the peripheral terminals of PSNs contributing to the onset of pain and inflammation but it also blocks the axoplasmic transport of these and other neuropeptides [93], thereby depleting terminals of their neuropeptide content. This block of nociception by the exhaustion of its main mediators at periphery was initially believed to be primarily responsible for pain relief after local capsaicin treatment. However it was more recently understood that the analgesic effect of capsaicin is mainly a consequence of a loss of function of the somatic nociceptive fibers [94]. In fact, the capsaicin-induced defunctionalization of these fibers not only derives from the depletion of neuropeptides but also from the loss of membrane potential, the block of neurotrophic factor axonal trafficking, and a reversible retraction of cutaneous terminals [94]. Nerve endings collapse because of the calcium overload induced by activation of TRPV1, with loss of mitochondrial function, metabolic inhibition and disruption of the integrity of the terminal membrane [94]. These observations are fully in line with previous studies on TRPV1-bearing cells *in vitro* that, after challenge with nontoxic doses of capsaicinoids, RTX or olvanil, downregulated their TRPV1 expression and ultimately underwent cell death [95,96]. In keeping with these *in vitro* studies, RTX causes the loss of unmyelinated C fibers and a significant damage to myelinated Aδ fibers in adult rats. The agonist also depletes TRPV1 expressing neurons in DRGs with reduced thermal pain perception [97]. Therefore, the defunctionalization that follows the activation of TRPV1 by capsaicin and its agonists/activators causes long-term functional and structural alterations that are not limited at the peripheral terminals of the PSNs.

Finally, it needs to be mentioned that repeated topical application of capsaicin also leads to degeneration of the cutaneous autonomic nerve fibers [98]. As a result, there is a reduction of pain by a still not clearly understood additional mechanism that, likely involves a TRPV1-mediated calcium influx and glutamate release from these fibers [99].

#### 2.2.2. Somatic Pathways

##### First-to-Second Order Neuron Synapses and Modulation in Substantia Gelatinosa

The painful effects of the administration of capsaicin to the skin occur very soon after an intradermal injection of the vanilloid, but the intensity of pain decreases progressively within twenty minutes. At the site of injection, the desensitization or defunctionalization of TRPV1-expressing primary afferents (see Section 2.1.1) results in a region of hypoalgesia. However, near the injection site, primary mechanical and heat hyperalgesia rapidly develop and last for about one day. It is widely agreed that primary mechanical and heat hyperalgesia are a consequence of the sensitization of PSNs [100]. Secondary mechanical hyperalgesia and allodynia instead appear in a progressively larger skin area after about fifteen minutes, to then slowly decrease in a few hours (allodynia) or in about one day (hyperalgesia) [101]. PSNs innervating the skin in a region of secondary mechanical allodynia and hyperalgesia are not sensitized by the capsaicin injection and thus, have a normal level of excitability. Therefore, an enhanced responsiveness (central sensitization) of the second order nociceptive STT or trigeminothalamic tract neurons is responsible for secondary mechanical allodynia and hyperalgesia [100].

The burning pain or sometimes itch, produced by the activation of peripheral TRPV1s [102] follows the discharge in polymodal C and mechano-heat Aδ cutaneous nociceptors. As mentioned in Section 2.2.1, these PSNs release a cocktail of neuropeptides at periphery. However, in the excited state, the central terminals of the PSNs release the same cocktail of neuropeptides and the fast amino acid transmitter glutamate in the spinal cord dorsal horn or the SNTN. These neurotransmitters act onto two main targets: the second order sensory STT projection neurons and the interneurons of the dorsal horn substantia gelatinosa [103]. Analogous effects are elicited onto the second order sensory trigeminothalamic tract neurons and the interneurons of the SNTN substantia gelatinosa. Notably, it was also the GABAergic interneurons to be affected by a topical injection of capsaicin into the skin, as demonstrated *in vivo* by the increased expression of proto-oncogene c-Fos in GABA-immunoreactive (IR) neurons following a challenge with the vanilloid [104].

##### Second-to-Third Order Neuron Synapses and Other Brain Areas Involved in Pain Control

In comparison to the enormous mass of data on first-to-second order neuron synapses, few studies have addressed the distribution and physiology of TRPV1 in supraspinal centers and more specifically, in STT synapses. Among the several areas of the brain that are directly or indirectly involved in the modulation of ascending nociceptive stimuli, TRPV1 has been localized in the somatosensory cortex, anterior cingulate cortex, insula, rostroventral medulla, PAG, amygdala and NTS [52,105,106]. Functionally, the role of the receptor is not totally clear, as capsaicin microinjected in the ventrolateral PAG had both hyperalgesic [107] and antinociceptive effects in rats [108,109,110]. The mechanisms of TRPV1 activation, function and sensitization of hypothetical supraspinal synapses in health and pathological pain conditions are discussed in [111].

##### Activation of TRPV1 during Pain Perception

Several lines of evidence converge to demonstrate that TRPV1 has indeed a role in bona fide pain perception. In a mouse model of neuropathic pain, a TRPV1-dependent glutamate-mediated cross talk among the prelimbic and infralimbic cortex neurons and glia participates to the generation of pain. In addition, single-unit extracellular recordings *in vivo*, following electrical stimulation of the basolateral amygdala or application of pressure on the hind paw, showed increased excitatory pyramidal neuron activity in these cortical areas, which also contained higher levels of the endocannabinoid 2-arachidonoylglycerol [112]. In another study, N-arachidonoyl-serotonin, which is a hybrid TRPV1 antagonist and fatty acid amide hydrolase inhibitor, normalized the imbalance between excitatory and inhibitory responses in the medial prefrontal cortex neurons, resulting in pain inhibition [113].

#### 2.2.3. Visceral Pathways

Visceral pain represents a major clinical problem, yet far less is known about its mechanisms compared to somatic pain. Since viscera are not normally exposed to noxious heat or capsaicin, the presence of TRPV1 renders the visceral afferents sensitive to the mediators of inflammation, thus functionally serving as nociceptors. As it will be further discussed, TRPV1 expression is found widespread in visceral innervation of all body organs and, when upregulated, TRPV1 correlates significantly with the degree of visceral pain. The importance of TRPV1 in visceral innervation is also supported by the painful effects of capsaicin application in several animal models and reports of human studies.

##### *TRPV1 Expression and Function in the Urinary Tract—Upper Urinary Tract* 

TRPV1 immunoreactivity is present in nerve terminals that course both the mucosa and muscular layer of the renal pelvis [114], with the exception of the rat kidney parenchyma [114]. TRPV1 may have a protective role in situations of exaggerated renal function and structural injury by attenuating the progression of renal fibrosis, possibly through down-regulation of the TGF-β/Smad2/3 signaling pathway [115]. In addition, TRPV1 attenuates renal inflammatory responses in mice subjected to DOCA-salt hypertension [116]. In the ureters, TRPV1 is detected in nerve terminals that course both the mucosa and muscular layer [114], where CGRP released from capsaicin-sensitive fibers acts as inhibitory transmitter, contributing to ureteral motility [117,118]. However, capsaicin also induces an inflammatory response in the ureters [45].

##### *TRPV1 Expression and Function in the Urinary Tract—Lower Urinary Tract* 

In the lower urinary tract, TRPV1 expression is well documented not only in a large subpopulation of nerve fibers but also in non-neuronal cells [114]. In the rat bladder mucosa, most IR fibers are in close proximity to the basal cells of the transitional epithelium, while in the vesical muscular layer TRPV1-IR fibers impinge on the surface of the smooth muscle cells. Functional TRPV1 channels in urothelial cells, including the basal, intermediate and large superficial umbrella cells, have been confirmed in rodents by either RT-PCR or immunolabeling [9,119]. In the human bladder, TRPV1-IR was detected in nerve fibers coursing in the suburothelial connective tissue and in muscular layer [120,121,122]. Charrua *et al.* confirmed the presence of TRPV1 channels in human urothelial cells by measuring TRPV1 mRNA levels in tissue extracts [123]. TRPV1-IR has also been reported in the interstitial cells of the human bladder [124] which form a suburothelial network that may contribute to a fast spread of smooth muscle contractions [125]; in the smooth muscle cells; endothelium of capillaries and arteries (but not of veins); and mast cells [120].

**Figure 2:**
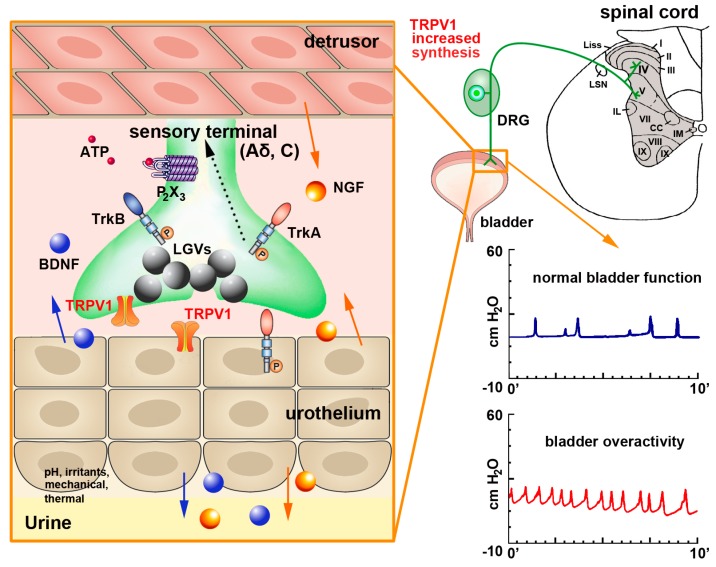
Schematics of TRPV1 localization and function in the urinary bladder and its contribution to bladder dysfunction. In the urinary bladder, TRPV1 can be found in sensory afferents and in the urothelium. Upon mechanical injury or inflammation TRPV1 levels are increased together with those of substance P and CGRP (stored in large granular vesicles—LGVs—large gray spheres) Nerve growth factor (NGF—orange spheres) is also released by the detrusor smooth muscle and the urothelium (orange arrows). NGF activates tropomyosin-related kinase A (TrkA) receptors expressed on afferent terminals, contributing to sensitization of neuronal TRPV1. The TrkA-NGF complex is internalized and retrogradely transported (dashed line) to neurons in lumbosacral dorsal root ganglia (DRGs), where de novo transcription of TRPV1 and additional sensory ion channels (including purinergic P_2_X_3_ receptor for ATP—small red spheres) is initiated. These newly synthesized ion channels are anterogradely transported back to afferent terminals to contribute to peripheral hypersensitivity. The urothelium also potentially produces brain-derived nerve factor (BDNF—blue spheres), which binds to tropomyosin-related kinase B (TrkB) receptors, further contributing to sensitization. This also participates to the development of bladder over activity, as shown by the increase number of bladder contractions in the graph at bottom right of the figure.

Given its extensive distribution, TRPV1 is important in regulating normal lower urinary tract function, and studies involving the desensitization of bladder sensory fibers by capsaicin and RTX have contributed to elucidate its mechanisms of action [126,127,128,129]. During bladder inflammation or spinal cord injury, TRPV1, substance P and CGRP expression is elevated [130] and this allows for the development of bladder overactivity and pain [126,129,131,132,133] (Figure 2). Pretreatment of rats with SDZ 249-665, a vanilloid compound reproducing capsaicin desensitization, attenuates inflammatory bladder hyperreflexia, referred hyperalgesia [134] and behavioral pain responses to intraperitoneal acetic acid in rats [135]. In mice with lipopolysaccharide (LPS)-induced bladder inflammation, an increase of the pain-evoked *fos* gene expression in sacral spinal cord neurons of wild type (WT) but not TRPV1 knock-out (KO) mice is observed, an effect that was accompanied by an increase of bladder reflex contractions [126,132,136]. Specifically, TRPV1 KO mice display greater short-term voluntary urination and abnormal urodynamic responses, with an increase in the frequency of non-voiding contractions, increased bladder capacity and inefficient voiding [136].

However, in the rat the density of TRPV1-IR fibers coursing in the mucosa or in the muscular layer was shown to be rapidly and markedly reduced after application of capsaicin or RTX [114,137] or even after TRPV1 antagonists [57,126,132,138], which significantly enabled an improvement of bladder function after inflammation or spinal cord injury. Similar findings were reported after intra vesical RTX application in patients with neurogenic detrusor over activity [121,139].

TRPV1 is also localized in nerve terminals within the mucosa and muscular layer of the urethra [114,122]. In keeping with this observation, application of capsaicin produced an inhibitory effect on the nerve-mediated contractions of the rat proximal urethra, promoting its relaxation and thus, facilitating urine voiding [140]. However, capsaicin also induces an urethral inflammatory response [141].

##### *TRPV1 Expression and Function in the Digestive Apparatus* 

TRPV1 in the digestive apparatus has been well documented in the afferents originating from PSNs in the DRGs, TG and nodose ganglion [142,143]. In general terms, TRPV1-IR fibers play an important role in modulating several physiological gut functions including motility, secretion, circulation and nociception [144,145]. However, TRPV1 is also emerging as a potential mechanosensor that mediates pain in various GI diseases [136,146,147].

##### Esophagus

Retrograde tracing and multiple labeling immunofluorescence studies have revealed that TRPV1-IR neurons projecting to the rat esophagus are located in the DRGs and the nodose ganglion [148]. TRPV1-IR nerve fibers deriving from these neurons are found in both the submucosal and myenteric plexuses of the esophagus. These fibers also express CGRP/substance P and neuronal nitric oxide synthase (nNOS) in the myenteric plexus of mice [149], and can be activated by capsaicin [150]. After exposure of the rat esophagus to acids, TRPV1 increases in DRGs [148,151]. In rats with chronic esophagitis, muscle afferent fibers exhibit a significantly greater response to capsaicin compared to non-inflamed naive animals [152]. This sensitized response is possibly due to an increase in the expression of TRPV1 channels in vagal afferent fibers after esophagitis. Activation of cervical and thoracic DRG neurons by intra-esophageal acid *in vivo* is lost in TRPV1 KO mice, suggesting that TRPV1 channels are required for acid-induced esophageal pain [153]. In keeping with this observation, TRPV1 KO mice develop a significantly less severe esophagitis after acid exposure [154]. Moreover, capsaicin causes enhanced relaxation of smooth muscle contraction in the esophagitis model, which supports the fact that acid reflux increases the expression of TRPV1 in primary afferent nerves [149]. In TRPV1 deficient mice, reflux promoting surgery induces significantly less mucosal injury and inflammation than in WT mice and pretreatment with the TRPV1 antagonist capsazepine in WT mice significantly inhibits esophagitis [152]. In cat spinal afferents, capsaicin-evoked stimulation of epithelial TRPV1 induces hypersensitivity and esophagitis due to the release of substance P and CGRP from esophageal sensory nerve endings [155].

Expression of TRPV1 is up-regulated also in human patients with esophagitis, gastro-esophageal reflux disease and non-erosive reflux disease [156,157,158]. The increase in the expression of TRPV1 correlates to intra-esophageal up regulation of nerve growth factor (NGF) and glial-cell-derived neurotrophic factor (GDNF) [157], which are important mediators involved in neuroplastic events and, therefore, contribute to enhancement of sensitization of the esophageal nerve fiber network.

##### Stomach

In rodents, most stomach-innervating neurons in nodose ganglia and DRGs have been shown to be IR for TRPV1 (80% and 71%, respectively) [142,151]. Furthermore, numerous TRPV1-IR nerve fibers were detected in the myenteric plexus, at the level of the fundus and the antrum. These fibers are in close contact with the myenteric ganglionic neurons. TRPV1-IR fibers were also observed within the circular and longitudinal muscle layers and at the level of the lamina propria of the mucosa. In these locations, TRPV1 has been found to co-localize with CGRP and substance P [159,160]. In humans, TRPV1 labeling was found in intra-cytoplasmic granules of the glandular parietal cells, in the gastric cells, in intramural ganglionic neurons and fibers, the latter being numerous in the submucosa and mucosa and, often, ending close to the parietal cells [161]. In addition, expression of TRPV1 protein and mRNA in a rat gastric mucosal epithelial cell line as well as in the mucosa of the intact rat stomach by Western blotting and RT–PCR, respectively, suggest that TRPV1 plays a protective role in these cells [162].

However, acute exposure of the rat gastric mucosa to a noxious HCl concentration has been shown to raise TRPV1 protein but not mRNA in DRG neurons innervating the stomach [151]. Capsaicin pre-treatment of rats prevents the behavioral pain reaction to gastric acid challenge [163]. In addition, central injection of capsaicin and anandamide stimulates gastric acid secretion in rats, via TRPV1 coupled with non-NMDA and GABA_A_ receptor systems [164,165].

In humans, the intragastric administration of capsaicin increases the sensitivity to proximal gastric distension [166], and ingestion of capsaicin capsules induces gastric sensations of pressure, heartburn and heat [167].

##### Small Intestine

Intense labeling of TRPV1-IR nerve fibers is found surrounding the myenteric neurons within the enteric ganglia of jejunum and ileum. Fibers can be found also in interganglionic tracts and they diverge within myenteric ganglia to wrap around cell bodies of the myenteric neurons [143]. Occasionally, TRPV1-IR nerve fibers can also be located within the circular muscle layer, surrounding the enteric neurons, and the blood vessels of the submucosal plexus. Retrograde labeling and immunofluorescence studies in DRGs revealed high degrees of co-localization between TRPV1 and CGRP, substance P or nNOS, in neurons supplying the mouse jejunum [168]. TRPV1-IR nerve fibers in the rat jejunum derive from extrinsic neurons, and activation of TRPV1 produces a relaxation response that is in part, due to the release of CGRP [169].

In general, capsaicin stimulates, most likely via TRPV1, the extrinsic afferents of the gut [150] and its administration into the lumen of the alimentary canal evokes pain in mice [170]. Capsaicin is thought to evoke intestinal pain by stimulation of jejunal chemoreceptors, presumably expressing TRPV1 [171]. Afferent jejunal nerve fibers can be activated by capsaicin, but this effect is obviously lost in TRPV1 KO mice [147]. Patients with uninvestigated dyspepsia have been found hypersensitive to intrajejunal capsaicin infusion [172]. In addition, administration of capsaicin into the ileum of patients with an ileal stoma has been reported to cause mechanical hypersensitivity [173].

##### Large Intestine

About three quarters of the colonic splanchnic afferents express TRPV1 and show capsaicin responsiveness [174]. Notably, the TRPV1-IR area in the rectum is the largest in the isolated mouse lower GI tract. Numerous TRPV1-IR nerve fibers were found in the mucosa, submucosal layer (around blood vessels), in the myenteric plexus and in the circular and longitudinal muscle layer; these fibers were found to contain CGRP, substance P and nNOS [175]. In the distal colon, TRPV1 fibers were half of that in the rectum, and in the transverse and proximal colon their density was even lower [175,176,177].

During pathological conditions the levels of TRPV1 increase in colonic tissues of rodents and humans, in parallel with a hypersensitivity of the large intestine [146,178,179,180] that could be inhibited by antagonist administration [181,182]. Rats pretreated with capsaicin do not present the inflammation-induced hypersensitivity that follows experimental colonic distension [183,184]. In a rat model of dextran sulfate sodium (DSS) colitis, neonatal animals chemically deprived of TRPV1-expressing fibers by treatment with capsaicin, as well as those given a TRPV1 antagonist (JNJ 10185734) were both protected from the damaging effects of DSS [179]. In keeping with these observations, Jones *et al.* found that the zymosan-induced sensitization of colonic afferents was absent in TRPV1 KO mice [146]. A mouse post-inflammatory chronic hypersensitivity model using intracolonic trinitrobenzene sulphonic acid (TNBS), which induces colitis, also showed TRPV1 as an important mediator of mechanical and chemical visceral hyperalgesia [182,185]. Furthermore, several studies have reported elevated expression of TRPV1-IR nerve fibers in large intestine biopsies from patients with irritable bowel syndrome and painful Crohn’s disease [25,180,186], Hirschprung disease [187], rectal hypersensitivity and fecal urgency [180]. Also, the density of TRPV1-positive nerve fibers in the rectosigmoid colon correlates with pain severity in patients with irritable bowel syndrome [186].

##### Pancreas

Through retrograde labeling of pancreatic nerves and immunostaining, it has been possible to localize the pancreatic afferents expressing TRPV1 in the mouse T9-T12 DRGs (75% of neurons) and the nodose ganglion (35% of the neurons) [188]. Within the glandular parenchyma, TRPV1-IR nerve fibers originating from these neurons are in close proximity to pancreatic acini [189]. Also in pancreatic nerve fibers, TRPV1, CGRP and substance P were found to be co-expressed. Capsaicin injection into the pancreatic duct promotes substance P and CGRP release in the dorsal horn neurons upon stimulation of pancreatic sensory nerves [189,190,191]. In turn, substance P has been shown to stimulate plasma extravasation from pancreatic post capillary venules [192]. In keeping with this observation, blocking substance P with specific NK1 receptor antagonists, or genetic deletion of the NK1 receptor, reduces pancreatic edema and neutrophil infiltration [192,193].

Capsaicinized animals incur in a much less severe pancreatic inflammation compared to non-capsaicinized animals, as the capsaicin-sensitive neurons expressing TRPV1 were destroyed [194]. However, administration of capsazepine significantly reduces inflammation and pancreatic injury in a model of caerulein-induced acute pancreatitis [195]. Injection of capsaicin into the pancreatic duct of rats has also been shown to induce expression of *fos* in the neurons of the spinal dorsal horn, contributing to pancreatic pain [196].

##### *TRPV1 Expression and Function in the Respiratory Tract* 

Fine axons expressing TRPV1 are diffusely distributed in the respiratory tract, specifically within the epithelium of the respiratory organs, including the trachea, bronchi, bronchioles and pulmonary alveoli. In these locations, TRPV1-IR fibers are also seen below the epithelium, surrounding the smooth muscles and blood vessels and around the alveolar wall [197]. Furthermore, most TRPV1-IR axons within the intrapulmonary airways co-express substance P and CGRP within and beneath the epithelium, around blood vessels, within airway smooth muscle and alveoli. However, only a small proportion of the nerves in the tracheal epithelium and only half the number of TRPV1 axons are immunopositive for substance P and CGRP [198].

During inflammation, lipoxygenase products, such as 15-HPETE, 15-HETE and leukotriene B4 (LTB4) released from the airways epithelial cells can directly activate TRPV1, thus contributing to neuronal hypersensitivity [199]. Increased production of NGF during asthma can potentiate inflammation through TRPV1 sensitization. In addition to these inflammatory mediators, acidic pH associated with inflammation can contribute further to TRPV1-mediated airway hypersensitivity during asthma [200].

##### *TRPV1 Expression and Function in the Genital Tract* 

In the male genital tract, rat TRPV1 mRNA is detected in the testicles, prostate and penis [201]. Similarly, transcripts for human TRPV1 were isolated from the testicles, seminiferous tubules, corpus cavernosum, glans and its overlying skin, scrotal skin and prostate [201]. In addition, TRPV1 could be found in Sertoli cells, where it regulated the acidity of extracellular microenvironment, which is crucial to maintain male fertility [202]. TRPV1 KO mice present testicular hyperthermia, which results in massive germ cell depletion from the seminiferous tubules [203]. In the human prostate, TRPV1 has also been described in primary afferents that course the urethral mucosa, verumontanum, ejaculatory ducts and periurethral prostatic acini, both by immunocytochemistry and western blotting [204]. The rich TRPV1 sensory innervation found in the human prostate plays an important role in the development of chronic prostatitis (Chronic Prostatitis/Chronic Pelvic Pain Syndrome—CP/CPPS). Burning pain sensation is the main description of pain in patients with CP/CPPS, either upon urination or ejaculation and is directly related to activation of TRPV1-IR fibers [204,205]. In addition, CP/CPPS patients have increased heat sensitivity in the perineal area [206]. TRPV1 immunoreactivity is also found in the epithelial cells of the prostate [207], where application of capsaicin and RTX causes a calcium inflow, revertible by capsazepine [208]. Notably, although low levels of TRPV1 mRNA are detected in the prostate, high levels of the protein occur in both the epithelium and smooth muscle cells of the gland [209].

In the female genital tract, TRPV1 is expressed in the vagina [210] and myometrium of the uterus. Rat uterine cervical afferents in the hypogastric nerve express TRPV1 [211] and estrogens amplify the responses to painful stimuli of the uterine cervix due to an increase of TRPV1 expression in PSNs innervating the uterus [212]. On the other hand, the activation of nerve fibers in the uterine horn is reversed by intrauterine pretreatment with capsazepine [212,213].

In a study investigating TRPV1 innervation of the human uterus during pregnancy and labor, TRPV1-IR fibers were observed scattered throughout the stroma and around blood vessels and appeared more frequently in the subepithelium of the cervix uterus. An almost complete disappearance of TRPV1-IR fibers was observed in the pregnant uterus, but the cervical innervation remained high throughout pregnancy and labor and was likely responsible for pain during cervical ripening [214]. In addition, TRPV1-expressing fibers significantly increased in the vulvar epidermis and superficial dermis [23].

## 3. Experimental Modeling Nociception Using Capsaicin *in Vitro* and *ex Vivo*

Under normal conditions *in vivo*, capsaicin and capsaicinoids only have access to cutaneous and mucosal TRPV1-expressing sensory fibers of the digestive tract, the airways and the conjunctiva. However, given the widespread expression of the receptor in tissues and organs, the molecule has been used as an agonist of TRPV1 in numerous *in vitro* and *ex vivo* studies. Most studies on isolated cells have been fundamental to understand the biology and function of TRPV1 as summarized in Section 1.3.2 and Section 2.1. Other studies *in vitro* led to a better understanding of the cellular pathways that are activated following receptor activation by the vanilloid.

It is well established that assessment of functional activity of TRPV1 can be evaluated by quantification of ATP release by capsaicin stimulation. In urothelial cells, this process was shown to be potentiated by NGF treatment and dependent on TrkA activation via phosphatidylinositol-3-kinase (PI3K) and protein kinase C signaling [215]. The relation between TRPV1 channels and NGF-induced pain is well established in somatic and visceral pain, and studies in heterologous expression systems contributed to demonstrate the biochemical mechanism through which bradykinin and NGF produce hypersensitivity and might explain how the activation of PLC regulates the activity of other members of the TRP channel family in PSNs [81].

*Ex vivo* systems are less commonly in use but they are very promising as a bridge, linking intracellular pathways studies and functional anatomy. In general, these preparations include at least bundles of primary fibers and a target organ, such as the intestine or the skin. For example, using an *ex vivo* colon-pelvic nerve-L6 DRG-and spinal cord preparation, mechanically sensitive colonic afferents were shown to segregate into high firing and low-firing frequency fibers based on firing frequency and distension response thresholds. Notably, nearly all low frequency afferents expressed TRPV1 and could be sensitized by luminal application of capsaicin, in contrast to the high-frequency ones [178].

Using acute jejunal preparations, capsaicin induced typical excitatory responses on sensory afferents of WT mice, which lasted briefly and were followed by desensitization with higher concentration of capsaicin. In contrast, capsaicin had neither excitatory nor desensitizing effects on jejunal nerves of TRPV1 KO mice [147].

Finally, capsaicin-evoked action potentials on isolated skin-nerve preparations were significantly decreased in cannabinoid 1 receptor KO mice [216].

**Figure 3:**
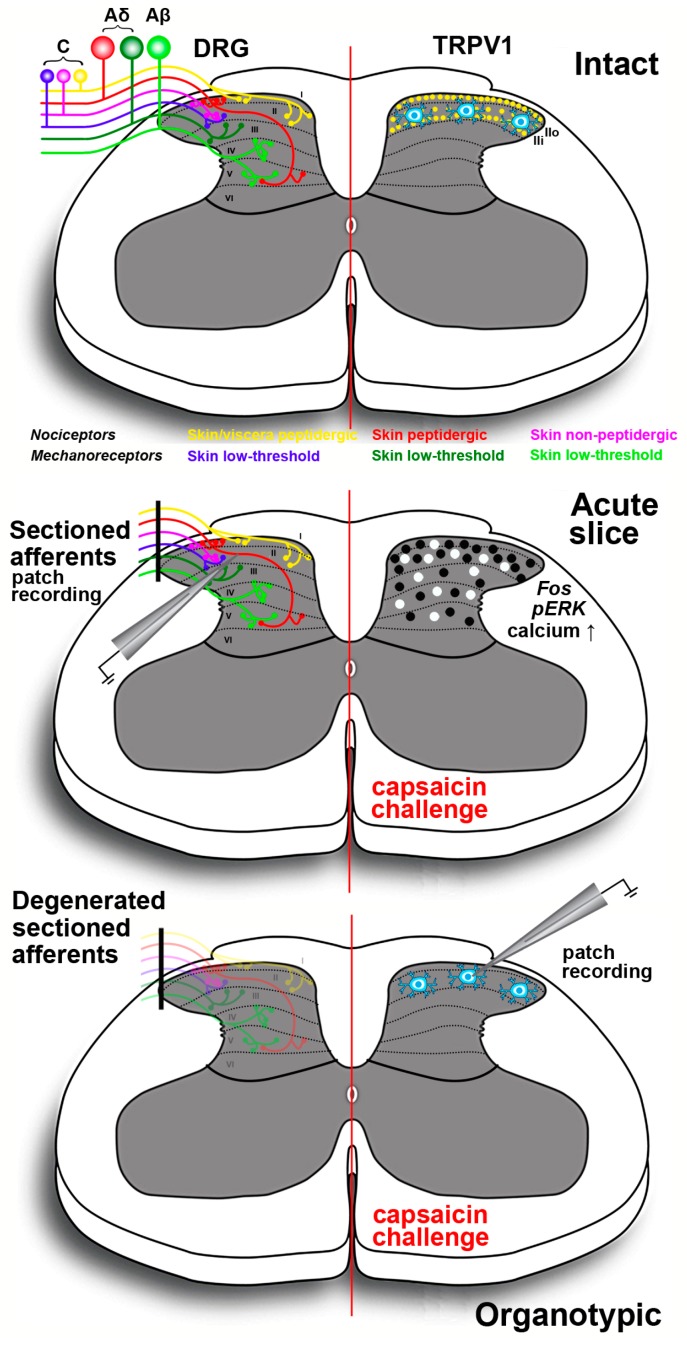
Schematic drawing of the spinal cord slices preparations exploiting the agonist action of capsaicin onto TRPV1 expressing neurons and fibers in the dorsal horn. Top panel shows at left the laminar distribution of primary afferent terminals originating from different classes of nociceptors in DRG neurons; at right the distribution of TRPV1 in peptidergic terminals (yellow dots) originating from skin and viscera in laminae I-II. Receptors are less densely localized in the outer part of lamina II (II_o_) as compared to lamina I and the inner part of lamina II (II_i_). Lamina II also contains TRPV1-IR interneurons that are spared in organotypically cultured slices (bottom panel). For simplicity, proprioceptive muscle afferents are not represented. In acute slices (middle panel) primary afferents are severed at dorsal roots but their terminals remain functional for several hours allowing performing electrophysiological recording, calcium imaging and proto-oncogene c-Fos/pERK immunocytochemistry. The black dots indicate the distribution of proto-oncogene c-Fos/pERK-IR neurons after capsaicin challenge. White dots exemplify the location of the cells responding to the vanilloid with increased intracellular calcium concentration in real-time confocal imaging. In organotypic cultures (bottom panel) sectioned primary afferents degenerate and thus, only the TRPV1-IR lamina II neurons are spared. This type of preparation is useful to isolate the effects of TRPV1 activation on these neurons, in the absence of primary afferent input.

Spinal cord slices (Figure 3) are another type of *ex vivo* preparation that proved to be useful for a better understanding of the central modulatory effects of TRPV1. Most diffuse are acute spinal cord slices, at times with attached dorsal roots and/or DRGs. However, the spinal cord was also organotypically cultivated thereby leading to deafferentation and disappearance of the primary sensory input to the spinal cord dorsal horn [217]. Initial studies on acute spinal cord slices were carried out with an electrophysiological approach and were focused onto lamina II, given its pivotal role in the modulation of nociception. Experiments aiming to establish whether the central and peripheral effects of capsaicin onto the internalization of the preferred substance P receptor NK1 had different mechanisms led to the demonstration that capsaicin produced neurokinin release by a direct action, *i.e.*, a TRPV1-mediated influx of Ca^2+^, on primary afferents terminals. The vanilloid also increased the firing of action potentials, and the first effect but not the second largely bypassed NMDA and GABA_B_ modulatory mechanisms [218]. These observations were consistent with the previous demonstration that capsaicin induced a strong rise of [Ca^2+^]_i_ in rat spinal cord slices during the course of laser scanning confocal microscope imaging experiments [219]. Converging to the demonstration of a cross-talk between central TRPV1 and peptide release, N-(4-tertiarybutylphenyl)-4-(3-cholorphyridin-2-yl)tetrahydropryazine-1(2H)-carbox-amide, a selective TRPV1 antagonist, inhibited the capsaicin-induced release of CGRP and substance P in rat spinal cord slices [220]. Subsequent electrophysiological observations showed that primary afferent stimulation with capsaicin differentially potentiated excitatory and inhibitory inputs to spinal lamina II outer and inner neurons [221]. Moreover, in another study, administration of capsaicin to lamina II neurons in slices from mice pups resulted in an increase of spontaneous inhibitory postsynaptic currents. GABAergic inhibitory interneurons in laminae I, III and IV were excited because of the release of substance P, which, in turn, reduced the activity of lamina II neurons [14]. Experiments on lamina I neurons also demonstrated that purinergic P_2_X receptor-expressing fibers were capsaicin-sensitive nociceptive afferents [222].

Other investigators used a different approach exploiting the use of capsaicin as a tool to stimulate peptidergic primary afferents in acute slices. This approach was based on the immunocytochemical localization of pERK or expression of the proto-oncogene c-Fos. ERK phosphorylation is known to occur in central sensitization and capsaicin stimulation of C fibers induced eight to tenfold increase of pERK in superficial dorsal horn neurons [223]. The effect was similar to that obtained after direct electrical stimulation of these fibers or intraplantar injection of capsaicin in an intact animal. More recently, a study has compared the pERK and proto-oncogene c-Fos responses of mouse spinal cord slices subjected to octreotide administration. Octreotide is a synthetic antinociceptive analog of somatostatin, one of the neuropeptides involved in the negative modulation of pain signals in the dorsal horn. In acute slices, octreotide reduced the response to capsaicin as measured by expression of proto-oncogene c-Fos and pERK, and it was concluded that the use of Fos and pERK immunoreactivity *in vitro* was a valuable tool in investigating the activation of spinal nociceptive pathways and to test potentially antinociceptive molecules [224].

Other *ex vivo* approaches were based on the use of real-time confocal imaging of cell-permeant calcium indicators or voltage-sensitive dyes. The strong calcium response induced by capsaicin was used in acute rat or mouse slices to mimic the central effects of inflammation and to study the ying and yang of BDNF and GDNF in the modulation of the excitatory and inhibitory input to lamina II interneurons [225,226]. Finally, the neurotoxic effects of neonatal capsaicin were exploited to prepare slices from C fibers’ depleted rats and to study the effects of GABA and excitatory amino-acid receptors antagonists on the primary afferent excitatory input to the spinal dorsal horn, by imaging slices from young pups after anterograde labeling with a voltage-sensitive dye from the dorsal root attached to the spinal cord slice [227].

## 4. Therapeutic Use of Capsaicin 

Capsaicin has been used in several clinical settings as a topical medication to treat pain derived from different conditions. The USA regulatory authorities have approved capsaicin as an 8% dermal patch for treating local pain. These patches contain 640 mcg/cm^2^ synthetic capsaicin with a total dose of 179 mg in one patch [228]. Lower doses showed no clinical benefits [229,230,231] or only short-term efficacy [232,233]. In general, it seems that the vanilloid is effective in the treatment of neuropathic but not inflammatory pain. For example, capsaicin was not effective against inflammatory osteoarthritic pain [234], and a high-dose capsaicin patch was of no benefit in targeting persistent pain after inguinal herniorrhaphy [235]. However, instillation of 15 mg capsaicin (Anesiva 4975) immediately prior to wound closure displayed some efficacy in controlling pain after total knee arthroplasty [236], and the vanilloid reduced abdominal pain of patients affected by irritable bowel syndrome after oral administration [237]. Notably, zucapsaicin, a synthetic cis isomer of natural capsaicin [238] was shown to be therapeutically effective in relieving pain deriving from knee osteoarthritis. The mechanism of action and clinical indications of zucapsaicin are similar to that capsaicin, but zucapsaicin is better tolerated. Therefore, zucapsaicin could become a valuable drug for treating certain forms of inflammatory pain such as osteoarthritic or intestinal pain and headaches. 

An 8% capsaicin patch was reported to be safe and effective in controlling neuropathic pain resulting from several conditions, with clear improvement in pain attacks, sleep duration and quality of life [239]. Local pain and erythema were the commonest adverse effects in about 10% of the patients. Capsaicin patch preparations (NGX-4010 or Qutenza^®^) were also of benefit in post-herpetic neuralgia and painful HIV-associated distal sensory polyneuropathy, alone [239,240,241,242,243,244,245,246,247,248] or in association with 4% lidocaine topical preparations [249,250]. Notably, the outcomes of a recent interdisciplinary expert workshop lead to the conclusion that response rates in patients with or without lidocaine pretreatment were comparable [251]. According to Derry *et al.* 2013 [252], high-concentration topical capsaicin is similar to other therapies for chronic pain. However, the high cost of single and repeated applications suggest that such a therapy should be preferentially used when other available therapies have failed. Moreover, the same authors concluded that this type of treatment should not be used repeatedly without substantial documented pain relief as, even when efficacy is established, there are unknown risks of repeated application for long periods, especially on epidermal innervation. In addition, it should be mentioned that a revision of the Special Interest Group on Neuropathic Pain (NeuPSIG) recommendations for the pharmacotherapy of neuropathic pain, based on the results of a systematic review and meta-analysis of existing clinical data, only led to weak recommendation for the use and proposal as second line therapy of capsaicin high-concentration patches [253].

Injectable capsaicin preparations are also in the course of clinical evaluation. Adlea (ALGRX-4975) is an injectable highly purified form of capsaicin formulated for long lasting pain relief [254]. It is currently under investigation for treatment of intermetatarsal neuromas [255], lateral epicondylitis [256] and end stage osteoarthritis [257]. However, a recent study has tested the effects of intradermal capsaicin in healthy volunteers and led to the conclusion that an injection of capsaicin at different depths in the skin had effects on heart rate and blood pressure but not on pain [258]. Authors worried that their results might have implications for the pharmacology and analgesic predictive value of the model of intradermal capsaicin.

Palvanil (N-palmitoyl-vanillamide) is a non-pungent capsaicin- like compound found in low amounts in *Capsicum* plants [259]. Palvanil has slower kinetics of TRPV1 activation and is a stronger desensitizer of TRPV1 than capsaicin [259]. When administered systemically at analgesic doses in mice, it produced significantly fewer aversive effects on body temperature and bronchoconstriction as compared to capsaicin [260]. These observations have a potential for subsequent translational studies.

## Figures and Tables

**Table 1:** Natural activators/agonists of TRPV1 receptor. Some of these molecules not only act as receptor agonists but also as channel blockers. Abbreviations: DkTx = Double-knot toxin; HPETE = hydroperoxyeicosatetraenoic acid; NADA = arachidonoyl-dopamine; NGF = nerve growth factor; RTX = resinferatoxin; VaTx = vanillotoxins.

Activators of TRPV1 Receptor			Action	Refs
**Physical activators**		Depolarization (V_½_ ~ 0 mV at 35 °C)	activator	[59]
		Noxious heat (> 43 °C at pH 7.4)	activator	[59]
**Endogenous activators**	***Protons***	Mild acidification (extracellular H^+^ pEC_50_ 5.4 at 37 °C)	activator	[58,59,72]
	***Small molecules***	Adenosine and ATP	activator	[73]
		Polyamines	activator	[74]
	***Lipids, lipid metabolites or derivatives***	lipoxygenase products (12-HPETE, 15-HPETE)	agonist	[75]
		leukotriene B4		[76]
		5-(S)-hydroxyeicosatetraenoic acid		[59]
		NADA N-oleoyldopamine	channel blocker	[77]
		anandamide (arachidonoylethanolamide)	channel blocker	[78]
		prostaglandins	activator	[79]
	***Peptides, proteins and growth factors***	bradykinin	activator	[80,81,82]
		prokineticin	activator	[83]
		protein kinase C	activator	[84]
		NGF		[81]
**Exogenous activators**	***Plant products or derivatives***	RTX (active compound from the cactus *Euphorbia resinifera*)	agonist	[20]
		piperine (pungent component in black pepper)	agonist	[85]
		camphor (terpenoid extracted from *Cinnamomum camphora*)	agonist	[86]
	***Venoms***	from jellyfish (crude extracts from *Aiptasia pulchella*, *Cyanea capillata*, *Physalia physalis* and *Chironex fleckeri*)	agonist	[87]
		VaTx1-3 (Tarantulas’ toxins from *Psalmopoeus cambridgei*)	agonist	[88]
		DkTx (from the Chinese earth tiger tarantula *Chilobrachys guangxiensis*)	activator	[89]

## Abbreviations

The following abbreviations are used in this manuscript:

12-HPETE	12-hydroperoxyeicosatetraenoic acid
ATP	Adenosine triphosphate
CaMK II kinase	Calmodulin- dependent protein kinase II
CGRP	Calcitonin Gene-Related Peptide
CP/CPPS	Chronic Prostatitis/Chronic Pelvic Pain Syndrome
DNA	Deoxyribonucleic acid
DRG	Dorsal Root Ganglion
ENS	Enteric Nervous System
GABA	γ-Aminobutyric acid
GI tract	Gastrointestinal tract
HEK 293	Human Embryonic Kidney 293
IR	Immunoreactive
15-HPETE	15-hydroperoxyeicosatetraenoic acid
BDNF	Brain Derived Neurotrophic Factor
cDNA	complementary DNA
CNS	Central Nervous System
DkTx	Double-knot toxin
DOCA	Deoxycorticosterone acetate
DSS	Dextran sulfate sodium
FAF1	Fas-Associated Factor 1
GDNF	Glial cell-Derived Neurotrophic Factor
HCl	Acid chloride
ICC	Immunocytochemistry
KO	Knock-out
LB4	Leukotriene B4
LPS	Lipopolysaccharide
NADA	Arachidonoyl-dopamine
NGF	Nerve Growth Factor
NKA	Neurokinin A
NMDA receptor	N-methyl-D-aspartate receptor
NOS	Nitric Oxide Synthase
NTS	Nucleus Tractus Solitarius
PAG	Periaqueductal grey
Palvanil	N-palmitoyl-vanillamide
pERK	Phosphorylated Extracellular signal-Regulated Kinase
PI3K	Phosphatidylinositol-3-Kinase
PIP2	Phosphatidylinositol 4,5-bisphosphate
PKB/Akt	Protein Kinase B/Akt
PKC	Protein Kinase C
PNS	Peripheral Nervous System
PSNs	Primary Sensory Neurons
RT-PCR	Reverse Transcription-Polymerase Chain Reaction
RTX	Resinferatoxin
SNTN	Spinal Nucleus of the Trigeminal Nerve
STAT3	Signal Transducer and Activator of Transcription 3
STT	Spinothalamic Tract
TG	Trigeminal Ganglion
TNBS	Trinitrobenzene sulphonic acid
TrkA	Tropomyosin kinase A receptor
TRP	Transient Receptor Potential
TRPV1	Transient Receptor Potential cation channel Vanilloid subfamily member 1
VaTx	Vanillotoxins
VR1	Vanilloid Receptor subtype 1
WT	Wild Type
